# Cancer-Targeting Applications of Cell-Penetrating Peptides

**DOI:** 10.3390/ijms26010002

**Published:** 2024-12-24

**Authors:** Liliana Marisol Moreno-Vargas, Diego Prada-Gracia

**Affiliations:** Research Unit on Computational Biology and Drug Design, Children’s Hospital of Mexico Federico Gómez, Mexico City 06720, Mexico

**Keywords:** cell-penetrating peptides, cancer, targeted drug delivery, apoptosis inductors, cancer immunotherapy, fluorescent peptide dye, targeted cancer therapeutic peptides, multi resistance, selective membrane disruption, peptide receptor radionuclide therapy

## Abstract

Cell-penetrating peptides (CPPs) offer a unique and efficient mechanism for delivering therapeutic agents directly into cancer cells. These peptides can traverse cellular membranes, overcoming one of the critical barriers in drug delivery systems. In this review, we explore recent advancements in the application of CPPs for cancer treatment, focusing on mechanisms, delivery strategies, and clinical potential. The review highlights the use of CPP-drug conjugates, CPP-based vaccines, and their role in targeting and inhibiting tumor growth.

## 1. Introduction

Cancer remains one of the most challenging diseases to treat due to the inherent complexity of tumor growth, metastasis, and resistance to conventional therapies. While significant progress has been made in identifying tumor-specific cellular pathways that can be effectively targeted in vitro, achieving safe systemic delivery of these therapeutic agents has proven difficult. Challenges such as limited tissue penetration, toxicity, and reduced functional efficacy in vivo have hindered the translation of these therapies to clinical practice. However, recent developments in cell-penetrating peptides (CPPs) have opened new pathways for precisely targeting tumor cells, offering a promising solution to overcome these delivery barriers.

CPPs are short peptides, 4 to 40 amino acids long, with a net positive charge at physiological pH, allowing them to cross cellular membranes while preserving the cargo’s functionality. Since their discovery over 30 years ago [1,2], CPPs have been extensively studied for delivering diverse payloads such as proteins, nucleic acids, liposomes, nanoparticles, and other therapeutic agents [3,4]. In addition to functioning as delivery vectors, CPPs may also include functional motifs for more complex therapeutic roles [5,6,7].

CPPs exhibit significant sequence diversity, with cationic, anionic, or amphiphilic variants, influenced by their amino acid composition and three-dimensional (3D) structure. This diversity, together with external conditions, determines their mode of cellular entry and efficiency in delivering various cargoes, whether covalently or non-covalently attached. CPP uptake involves multiple pathways, including macropinocytosis, caveolae-mediated, and clathrin-dependent endocytosis [7,8,9,10]. Some CPPs can bypass these mechanisms and translocate directly across membranes, influenced by the cell’s lipid composition and the CPP’s physicochemical properties [11,12]. Some CPP’s uptake varies with concentration; at higher levels, they undergo direct translocation, while lower concentrations favor endocytosis [13,14,15]. Like many peptide-based therapeutics, CPPs face absortion, distribution, metabolism, excretion, and toxicity (ADMET) challenges, such as rapid renal clearance and low permeability [16]. Enhancing their pharmacological properties involves strategies like incorporating unnatural amino acids or associating CPPs with carrier proteins [15,17,18,19,20].

Despite these obstacles, CPPs offer great potential for targeted drug delivery due to their specificity, selectivity, and biocompatibility. Advances in CPP design, particularly in membrane translocation and cargo loading, continue to expand their therapeutic applications. In this sense, the use of CPPs in cancer research encompasses multiple strategies, with one particularly promising approach being their conjugation with cytotoxic drugs to improve tumor specificity [21,22].

In this review, we discuss the current landscape of CPP applications in cancer research and highlight promising preclinical and clinical studies.

## 2. Cell-Penetrating Peptides with Cancer-Targeting Applications

Recent advances in CPP technology, particularly the development of tumor-targeting CPPs, have significantly improved their ability to selectively recognize and penetrate cancer cells, reducing off-target interactions and minimizing systemic toxicity. These CPPs have demonstrated remarkable versatility in not only delivering therapeutic agents but also in modulating critical intracellular pathways involved in tumor growth and survival (bifunctional peptides). By facilitating precise drug delivery and selectively disrupting key protein interactions within cancer cells, these peptides offer a targeted approach that enhances therapeutic efficacy while reducing adverse effects. Moreover, CPPs have also been leveraged for diagnostic innovations, such as real-time tumor visualization, enabling improved surgical precision and treatment monitoring through advanced imaging techniques.

In the following subsections, we present a detailed overview of several CPPs that have shown successful applications in cancer research and clinical settings. Each example highlights unique mechanisms of action and clinical potential, illustrating the significant strides made in the field and reinforcing the promise of CPPs as pivotal agents in advancing cancer-specific therapies and diagnostics.

### 2.1. PEP-010: A Bifunctional Peptide Targeting Caspase-9/PP2A Interaction to Induce Apoptosis

PEP-010 is a bifunctional CPP specifically engineered to disrupt key intracellular interactions that are critical for tumor cell survival. In breast cancer models, PEP-010 targets the interaction between caspase-9 and protein phosphatase 2A (PP2A), with preclinical in vivo studies demonstrating significant tumor regression, underscoring its therapeutic potential for aggressive cancers [23,24].

**Figure 1:**
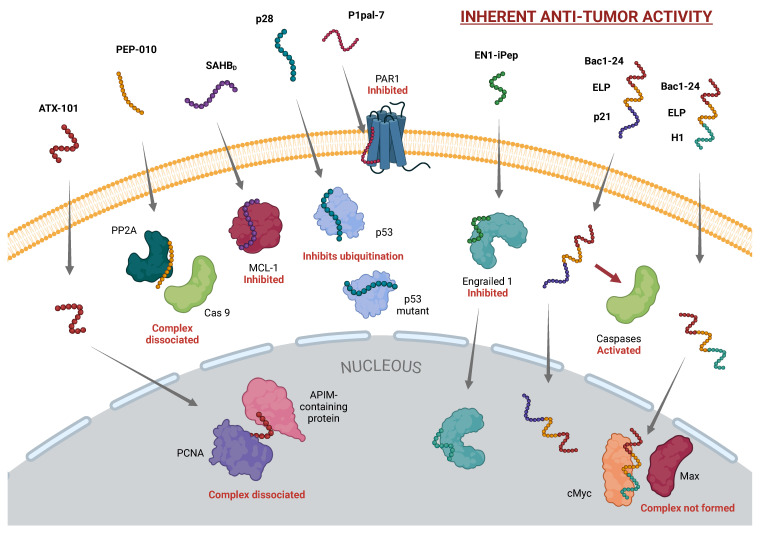
CPPs with Intrinsic Anticancer Activity. This figure presents CPPs that exert therapeutic effects through intrinsic biological functions, independent of cargo. Highlighted CPPs include ATX-101, which disrupts PCNA interactions; PEP-010, which induces caspase-9-mediated apoptosis; SAHBD, which inhibits MCL-1 to promote apoptosis; p28, stabilizing p53 for cell-cycle arrest; P1pal-7, which targets PAR1 to reduce tumor angiogenesis; EN1-iPEP, a transcription factor inhibitor triggering selective apoptosis; and Bac1-24, enhancing nuclear localization of therapeutic peptides. Only selected examples of the CPPs discussed in this communication are shown. [Created in BioRender. Moreno-Vargas, L. (2024) BioRender.com/c08a709 | CC-BY 4.0].

While the precise amino acid sequence remains proprietary for intellectual property protection, it is a peptide composed of 30 amino acids. This bifunctionality arises from two distinct sequences: the DPT domain, which ensures efficient cell membrane penetration, and the Pep-1 domain, which specifically interferes with PP2A [25]. Once inside the cell, PEP-010 disrupts the caspase-9/PP2A interaction (see Figure 1), reactivating caspase-9 and inducing apoptosis [23,26]. Caspase-9 activation subsequently initiates a cascade of caspase-dependent apoptosis, leading to tumor cell death. PP2A, a serine/threonine phosphatase, plays a pivotal role in regulating cell growth and DNA repair, making it an attractive target for cancer therapy [27].

This apoptotic restoration has been validated through both in vitro and in vivo studies, particularly in patient-derived xenograft (PDX) models of triple-negative breast cancer (TNBC) and hormone receptor-positive, HER2-negative breast adenocarcinoma [28] and ovarian carcinoma cells [29]. PEP-010 is currently undergoing its first clinical trial in humans (NCT04733027), a Phase I study aimed at evaluating its safety, tolerability, and optimal dosing, either as a monotherapy or in combination with chemotherapeutic agents like Paclitaxel and Gemcitabine. The data from this trial will offer critical insights into the pharmacodynamics, pharmacokinetics, and therapeutic potential of PEP-010 in cancer treatment [29].

### 2.2. ATX-101: A CPP Targeting Proliferating Cell Nuclear Antigen (PCNA) for Enhanced Cancer Therapy

Among the various CPPs under investigation, cationic CPPs (cCPPs) have shown promise due to their capacity to interact with negatively charged cell membranes, facilitating selective entry into tumor cells. One standout example in the field is ATX-101 (MDRWLVKWKKKRKIRRRRRRRRRRR), a CPP with unique structural and functional properties. ATX-101 is a cCPP composed of three functional components: an AlkB homolog 2 proliferating cell nuclear antigen (PCNA)-interacting motif (APIM; RWLVK), an SV40 nuclear localization signal (KKKRK), and the CPP undeca-arginine (R11) [30]. The APIM motif enables ATX-101 to interact with PCNA, a protein involved in various essential cellular processes, including stress responses, DNA damage repair, intracellular signaling, apoptosis, metabolism, and immune responses against tumors. By disrupting the PCNA/APIM-containing protein interactions (see Figure 1), ATX-101 enhances the efficacy of several anticancer agents, including DNA-damaging drugs, microtubule-targeting agents, molecular inhibitors (such as p38 MAPK and EGFR inhibitors), and γ-irradiation [31,32].

In a study by Müller et al., ATX-101 was shown to induce caspase-dependent apoptosis in multiple myeloma cell lines and primary cancer cells, independent of the cell cycle phase. Furthermore, the peptide increased the sensitivity of these cancer cells to melphalan, a DNA-damaging agent widely used in multiple myeloma treatment [30]. Preclinical research has confirmed that ATX-101 rapidly penetrates cells and targets PCNA/APIM-containing protein complexes, which are essential for cell survival and DNA repair mechanisms. A completed Phase I clinical trial (NCT01462786) demonstrated a favorable safety profile for ATX-101, with no significant adverse effects reported. This peptide is currently undergoing further evaluation in clinical trials. A Phase I/II trial is investigating its combination with platinum-based chemotherapy in patients with platinum-sensitive fallopian tube and primary peritoneal cancer (NCT04814875) [33]. Additionally, a Phase II study (NCT05116683) has been launched to assess the efficacy of ATX-101 as a monotherapy for sarcoma, focusing on its antitumor activity, safety, and pharmacokinetics.

### 2.3. AVB-620: A Novel Fluorescent Peptide Dye for In Vivo Malignant Tissue Visualization

**Figure 2:**
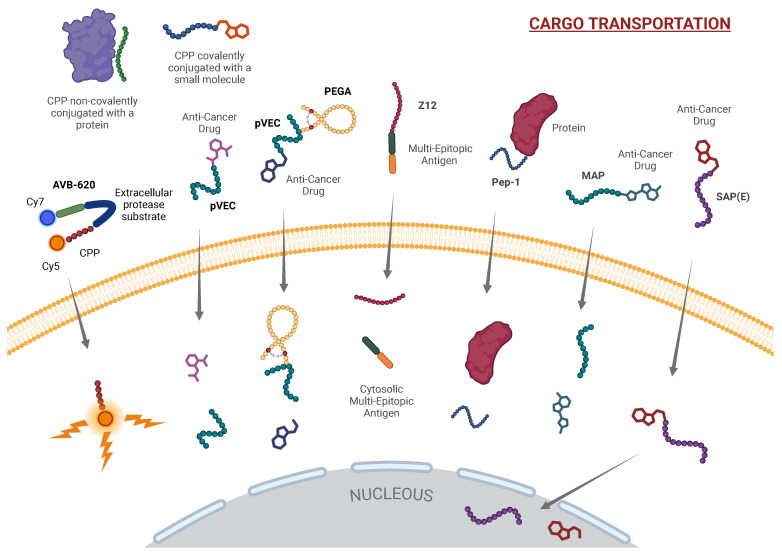
CPPs as Cargo Carriers for Targeted Delivery. This figure highlights the role of CPPs as efficient drug delivery vehicles, enabling targeted transportation of therapeutic agents into cancer cells. Included CPPs are AVB-620, pVEC, PEGA, Z12, Pep-1, MAP, and SAP(E), which exhibit diverse cargo-loading mechanisms: AVB-620 for real-time imaging, pVEC for direct cell translocation, PEGA for selective tumor targeting, Z12 and Pep-1 for immune modulation and selective cell penetration, MAP for membrane disruption, and SAP(E) as a complex-forming agent to enhance cellular uptake and cytotoxicity of its payloads in target cells, minimizing off-target toxicity. Only selected examples of the CPPs discussed in this communication are shown. [Created in BioRender. Moreno-Vargas, L. (2024) BioRender.com/u64r118 | CC-BY 4.0].

Another promising advancement in the application of peptides in cancer research is AVB-620, a protease-cleavable peptide designed for real-time tumor visualization during surgery. Unlike traditional CPPs used primarily for drug delivery, AVB-620 is engineered for diagnostic purposes. This peptide is conjugated with the fluorophores Cy5 and Cy7 at its cationic and anionic terminals, respectively, enabling fluorescence resonance energy transfer (FRET) between the two fluorophores, a process integral to its imaging capabilities. To enhance the solubility of AVB-620 in aqueous environments, an α-aminoxyl-ω-methoxy polyethylene glycol (mPEG) moiety (Mw 2000) was incorporated into its structure. This conjugation not only improves its water solubility but also stabilizes the peptide’s unique hairpin structure, optimizing FRET efficiency. The peptide is designed with a protease-cleavable linker that responds to matrix metalloproteinases (MMPs), particularly MMP2 and MMP9, which are overexpressed in human breast cancer cells. Upon cleavage by these MMPs, the Cy5 and Cy7 fluorophores produce a ratiometric fluorescence color change, which can be captured using specialized camera systems that concurrently record fluorescence and white light images [34,35,36] (see Figure 2).

This fluorescence-based imaging system significantly enhances the surgeon’s ability to visualize tumor tissue in real-time during operations, allowing for more precise resection of malignant tissues. In its first in-human Phase I clinical trial (NCT02391194), AVB-620 demonstrated both safety and effectiveness in detecting tumor-positive tissues during surgery [37]. This trial provided foundational data on the peptide’s potential to accurately differentiate malignant from non-malignant tissues during surgery.

Building on the Phase I results, a subsequent Phase II single-arm, open-label study (NCT03113825) was conducted to further evaluate AVB-620 in women undergoing surgery for primary breast cancer. This trial sought to determine how the timing of AVB-620 administration affects fluorescence imaging accuracy and its ability to reliably distinguish between malignant and non-malignant tissues intraoperatively. Results showed that AVB-620 allowed for real-time detection of tumor tissues, with improved imaging accuracy compared with conventional methods, facilitating more thorough excision of malignant tissues. AVB-620’s ability to provide surgeons with real-time visual feedback offers a significant leap forward in surgical oncology. This peptide’s capacity for selective activation in tumor environments, coupled with its real-time fluorescence imaging, opens new avenues for improving surgical outcomes. By enabling more precise excision of tumor tissues while sparing healthy tissue, AVB-620 holds the potential to minimize residual disease and reduce the need for re-operations, ultimately improving patient prognosis in breast cancer surgeries.

In summary, AVB-620 exemplifies the potential of protease-activated peptides in the surgical setting, demonstrating how innovations in peptide engineering can extend beyond therapeutic applications to also improve diagnostic precision. Ongoing studies will provide further insight into its efficacy across various cancer types and its potential integration into routine surgical practice.

### 2.4. Z12 and ZEBRA-Derived CPPs: Advancing Cancer Immunotherapy with Enhanced Vaccine Efficacy

A notable advancement in the field of cancer immunotherapy is the use of the Z12 peptide. This peptide (KRYKNRVASRKCRAKFKQLLQHYREVAAAKSSENDRLRLLLK) has been explored extensively for its role in the development of cellular-based vaccines by conjugating its sequence with multi-epitopic antigens [38]. These Z12-formulated vaccines have demonstrated the capacity to extend survival rates in preclinical tumor models, including a particularly aggressive brain cancer model, where the most pronounced survival benefits were observed.

The mechanism underlying Z12’s efficacy stems from its ability to modulate the immune response within the tumor microenvironment. In treated models, analysis of tumor sites revealed robust immune modulation, which not only promoted an integrated immune response but also facilitated the persistence and homing of CD8+ effector T cells to the tumor. This is crucial for maintaining long-term antitumor immunity. Additionally, the vaccines led to the activation of Th1-polarized CD4+ T cells, further contributing to potent antitumor responses in a variety of cancer models, including gliomas [38]. These findings highlight Z12’s potential as a versatile carrier for multi-epitope antigens, making it a promising candidate for the development of innovative cancer immunotherapies. Preclinical studies have demonstrated the peptide’s capacity to enhance the effectiveness of cancer vaccines by promoting immune cell infiltration into tumor sites and sustaining the antitumor activity over extended periods. In recognition of this potential, a Phase I clinical trial (NCT04046445) has been initiated to evaluate the safety, tolerability, and antitumor activity of Z12-based vaccines, specifically ATP128, VSV-GP128, and BI 754091, in patients with stage IV colorectal cancer. This trial marks a critical step toward translating Z12’s preclinical success into clinical applications, offering a promising outlook for human cancer immunotherapies [39].

Beyond Z12, considerable attention has also been directed toward other CPPs derived from the ZEBRA protein. These peptides, including truncated forms of ZEBRA, have been evaluated for their structural properties, ability to efficiently transduce cells, and their capacity to induce CD4+ and CD8+ T cell responses in vivo. Several studies have underscored the importance of selecting the appropriate CPP sequences and adjuvants to optimize antitumor immunity. For example, optimal combinations of CPPs and adjuvants have been shown to significantly enhance immune responses and control tumor growth, particularly in models of aggressive cancers [40,41,42].

Z12 and ZEBRA-derived CPPs have demonstrated notable potential as components of cancer vaccines. Their ability to induce potent immune responses—especially in combination with other adjuvants—positions them as valuable tools for advancing cancer therapies. These findings further validate the growing interest in utilizing CPPs not only for drug delivery but also for eliciting strong, targeted immune responses, offering new strategies for the development of therapeutic cancer vaccines.

### 2.5. pVEC: A Versatile Non-Endocytic CPP for Targeted Delivery of Therapeutic Biomolecules

Another noteworthy peptide in cancer research is pVEC, a CPP with distinct characteristics that make it an effective carrier for therapeutic biomolecules. The peptide sequence of pVEC (LLIILRRRIRKQAHAHSK) enables its internalization through a non-endocytic translocation mechanism, bypassing the need for endocytic pathways. Importantly, this mode of uptake does not alter the permeability of the plasma membrane or affect cell morphology, making pVEC a safe and efficient delivery vector for a variety of therapeutic agents, including peptide nucleic acids (PNAs) and proteins [43].

A series of studies aimed at understanding the functional domains of pVEC have highlighted the significance of the peptide’s N-terminal hydrophobic region in driving efficient cellular translocation. Specifically, single-residue substitutions within this hydrophobic region have been shown to markedly affect the peptide’s ability to translocate across cell membranes, underscoring the importance of sequence integrity for maintaining its translocation efficiency [44]. These findings suggest that careful sequence design is critical for optimizing pVEC’s role as a delivery vehicle in therapeutic applications.

Beyond its inherent ability to penetrate cells, pVEC has also been conjugated with homing peptides that selectively target molecular markers expressed on tumor cells. A notable example is the cyclic peptide PEGA (CPGPEGAGC), which has been shown to accumulate preferentially in breast tumor tissues in mouse models. In vitro experiments further demonstrate that PEGA, when conjugated to pVEC, is effectively internalized by various breast cancer cell lines, including MCF-7 cells [45]. This combination of pVEC and PEGA enhances the selectivity of drug delivery, ensuring that therapeutic agents are concentrated in tumor tissues (see Figure 2).

One of the most compelling applications of pVEC lies in its use as a delivery vehicle for anticancer drugs. For instance, when the anticancer drug chlorambucil was conjugated to pVEC-PEGA, the conjugate exhibited significantly improved drug efficacy. Specifically, the conjugated form of chlorambucil enhanced its cytotoxicity more than fourfold compared with the unconjugated drug, as evidenced by a marked reduction in clonogenic survival of MCF-7 breast cancer cells [46,47]. This result underscores the potential of pVEC as a carrier for chemotherapeutic agents, offering a more targeted and potent approach to cancer treatment.

The ability of pVEC to target nuclear structures after translocation further expands its utility, particularly for delivering nucleic acid-based therapeutics such as PNAs. Given the challenges associated with efficiently delivering PNAs to intracellular targets, pVEC’s capability to localize within nuclear compartments positions it as a valuable tool for gene modulation therapies. Moreover, the non-endocytic mechanism of pVEC’s uptake reduces the risk of endosomal entrapment, which is a common limitation in other CPP-based delivery systems [43,44].

### 2.6. Pep-1: Exploiting Membrane Composition for Enhanced Selectivity in Targeting Cancer Cells

**Figure 3:**
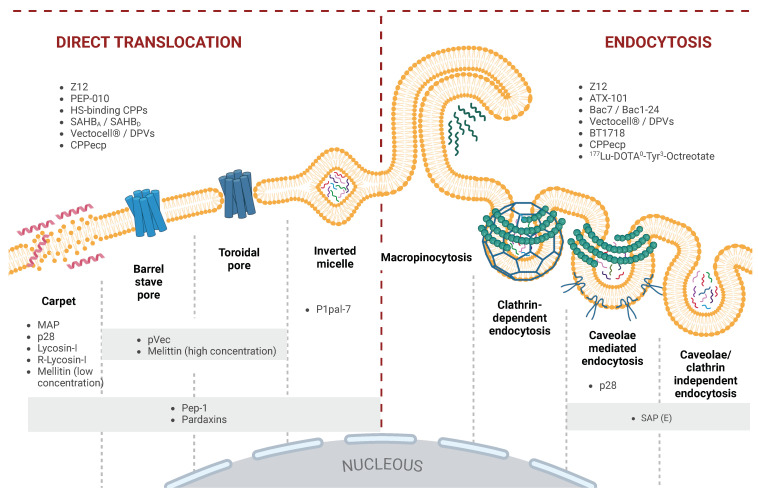
Internalization Mechanisms of CPPs Across Cell Membranes. CPPs employ diverse mechanisms to cross cell membranes, including direct translocation, endocytosis, and interactions with receptors overexpressed in membranes. Despite shared characteristics, CPPs exhibit distinct internalization routes that vary across peptide families and depend on experimental conditions. Factors such as charge, length, structure, and peptide concentration play critical roles in determining the internalization route. The CPPs listed in this communication are highlighted, noting that individual CPPs can often engage multiple internalization pathways. [Created in BioRender. Moreno-Vargas, L. (2024) BioRender.com/j68n394 | CC-BY 4.0].

One more important CPP, Pep-1, has garnered significant attention for its unique mechanism of membrane interaction and ability to deliver a broad range of macromolecules into cells. Upon contact with lipid membranes, Pep-1 undergoes a conformational rearrangement, which results in lipid segregation, membrane disorganization, and the transient formation of pores. These pores allow for ionic currents to pass through the membrane, facilitating the peptide’s internalization [48,49] (see Figure 3).

The initial interaction between Pep-1 and the membrane is primarily mediated by electrostatic forces, particularly between the positively charged hydrophilic domain of Pep-1 and the negatively charged polar head groups of membrane phospholipids. As Pep-1 inserts into the membrane, dehydration and subsequent embedding of its hydrophobic domains promote membrane destabilization, further enhancing its translocation across the lipid bilayer [50,51,52,53].

Pep-1 exhibits differential selectivity for cancer and normal cell membranes, which is influenced by the composition of these membranes. Cancer cell membranes typically exhibit a larger surface area due to microvilli formation, a higher net negative charge, and increased fluidity resulting from reduced cholesterol content [54]. Studies have shown that Pep-1 interacts more strongly with cancer cell membranes, primarily due to enhanced electrostatic interactions with the elevated acidic components present on the surface of tumor cells. In contrast, its interaction with normal cells is driven more by hydrophobic forces, particularly with the phosphate groups in the membrane [55,56]. This selectivity makes Pep-1 a promising candidate for targeting cancer cells while sparing normal tissues.

Moreover, the selectivity of Pep-1 is linked to variations in the levels of phosphatidylserine (PS) and other acidic components on the outer membrane of cancer cells compared with normal cells [57]. Such nuances in membrane composition allow Pep-1 to preferentially interact with cancerous tissues, highlighting its potential for selective therapeutic delivery.

While several studies have demonstrated that Pep-1 adopts different secondary structures upon interaction with membranes, the exact structural conformation required for efficient translocation remains unclear [58,59]. Despite this uncertainty, Pep-1 has shown remarkable versatility in its ability to transport a wide range of peptides and proteins into various cell types, all while maintaining the biological activity of the cargo. These include neuronal cells [60], pancreatic cells [61,62], neural retinal cells [63], macrophages [64], and hepatocytes [65].

A key advantage of Pep-1 lies in its ability to deliver active macromolecules without relying on the endosomal pathway, thereby bypassing one of the major limitations associated with endocytic uptake. This endosomal bypass prevents degradation of the cargo in lysosomes, ensuring efficient delivery of biologically active molecules to the cytoplasm or other intracellular compartments. Furthermore, dissociation of the Pep-1 macromolecule complex occurs almost immediately upon crossing the cell membrane, allowing the delivered molecules to exert their effects quickly and efficiently [66] (see Figure 2).

Henriques et al. explored the mechanism by which Pep-1 facilitates the translocation of β-galactosidase (β-Gal) across lipid vesicle membranes into HeLa cells. Their findings reveal that β-Gal translocates through a non-pore-forming, electrostatic pathway, bypassing endosomal and caveolin-mediated uptake, thus preserving cell viability. The β-Gal and Pep-1 complex was formed non-covalently, with Pep-1’s hydrophobic and basic residues driving electrostatic and hydrogen bonding interactions. This method maintained β-Gal’s enzymatic activity, enabling effective membrane translocation without loss of intracellular functionality [50]. Studies with Pep-1 have highlighted its potential for targeted delivery in cancer therapy applications by preserving the functional activity of therapeutic biomolecules, such as apoptosis-inducing proteins or small molecules, thus enhancing their effectiveness within the intracellular environment [67,68,69].

In 2019, Guo et al. conducted a study aimed at enhancing the targeted delivery of therapeutic agents to glioma cells. The primary objective of using Pep-1 in their research was to achieve tumor-targeted specificity by modifying carmustine-loaded micelles. Pep-1, a cell-penetrating peptide with high affinity for the IL-13R*α*2 receptor, overexpressed in glioma cells, facilitated selective internalization of the micelles via receptor-mediated uptake, bypassing nonspecific endocytic pathways. In addition to promoting selective internalization, Pep-1’s function was complemented by borneol, which facilitated blood–brain barrier penetration, resulting in efficient and sustained accumulation of the therapeutic agent in glioma tissues. This targeted approach enabled precise drug delivery directly into tumor tissue, thereby enhancing therapeutic efficacy and minimizing systemic side effects [70].

In addition to its high affinity for proteins and peptides at nanomolar concentrations, Pep-1 is stable in physiological buffers and exhibits no detectable toxicity, making it a particularly attractive candidate for therapeutic applications. Its non-toxic profile, coupled with its ability to form covalent protein transduction domains, places Pep-1 as a versatile tool in the development of therapeutic strategies aimed at delivering functional proteins, peptides, or other macromolecules into targeted cells.

In summary, Pep-1’s ability to selectively interact with tumor cell membranes, its efficient non-endocytic translocation, and its capacity to preserve the biological activity of a wide range of therapeutic cargoes underscore its significant potential in cancer therapy.

### 2.7. MAP: Amplifying Cytotoxicity and Antiproliferative Effects in Cancer Therapy Through Drug Conjugation

A further class of CPPs that has shown significant promise is the secondary amphipathic CPPs (saCPPs). These peptides, including the well-characterized Model Amphipathic Peptide (MAP), are designed with distinct hydrophobic and hydrophilic residues positioned on opposite sides of their helical structures, facilitating strong interactions with negatively charged phospholipids in cell membranes. The amphipathic α-helix of MAP (KLALKLALKALKAALKLA) enables it to effectively insert into lipid layers, where it disrupts membrane integrity [71].

Studies on the kinetics of cellular uptake for various CPPs have consistently demonstrated that MAP exhibits a rapid rate of uptake, accompanied by the ability to induce membrane leakage at low concentrations, as low as 1 µM. This membrane-disrupting activity is attributed to its robust interactions with the lipid bilayer, which compromises the structural integrity of the membrane. MAP’s capacity to alter membrane integrity also leads to its strong cytotoxic effects on a wide range of cell lines, further distinguishing it from other CPPs [72].

A compelling application of MAP was highlighted in recent research investigating its potential to enhance the antiproliferative properties of the drug Tacrine, an acetylcholinesterase inhibitor. When conjugated with Tacrine, MAP exhibited heightened toxicity against breast cancer (MCF-7) and neuroblastoma (SH-SY5Y) cell lines. Interestingly, both the conjugated and unconjugated forms of MAP displayed equivalent cytotoxic effects, suggesting that the peptide’s primary function in these models was more aligned with its role as a cell-killing agent rather than a traditional CPP [73].

In exploring MAP analogs with inverted charge distributions, such as MAP17 (QLALQLALQALQAALQLA), studies have revealed that the translocation ability of these peptides is closely tied to their amphiphilic nature. Like MAP, MAP17 possesses the structural properties necessary for membrane translocation, allowing it to interact efficiently with lipid bilayers. However, the inverted charge distribution may alter its specific interactions with cell membranes, potentially offering a different therapeutic profile compared with its parent peptide [74].

Ultimately, amphiphilic saCPPs like MAP offer a unique combination of rapid membrane translocation and potent cytotoxicity, making them valuable tools in cancer treatment. Their ability to disrupt cell membranes, coupled with their versatile structural properties, establishes them as potent agents for therapeutic interventions aimed at proliferative diseases. The continued development of these peptides, including further exploration of analogs like MAP17, holds the potential for significant advances in the field of peptide-based therapies.

### 2.8. p28: A Dual-Action CPP Targeting Wild-Type and Mutant p53 for Comprehensive Cancer Therapy

A different notable CPP that has gained increasing attention in cancer research is p28. This peptide, naturally derived from the bacterium *Pseudomonas aeruginosa,* serves both as an efficient CPP and a promising anticancer agent. Structurally, p28 is an amphipathic α-helical peptide composed of 28 amino acids (Leu50-Asp77), originating from azurin, a copper-binding redox protein of the cupredoxin family, known for its ability to modulate redox processes in cells [75]. The bifunctional role of p28 has sparked significant interest, particularly for its capability to penetrate various solid tumor cells [76].

The mechanism of action of p28 is distinctive and involves two major pathways. After internalization into the tumor cells, p28 binds to both wild-type and mutant forms of the tumor suppressor protein p53, a crucial regulator of cell cycle and apoptosis. This binding inhibits the ubiquitination of p53 by blocking the activity of constitutive photomorphogenesis 1 (Cop1), a protein that mediates p53 degradation via the proteasome. As a result, p28 stabilizes p53 levels, allowing it to accumulate within the cell. This accumulation of p53 induces G2/M phase cell-cycle arrest, followed by the activation of apoptotic pathways, thereby reducing tumor cell viability and promoting cell death [77].

Notably, this p53-targeting mechanism is particularly significant in cancer therapy, given that p53 is often mutated or dysregulated in many forms of cancer. By targeting both wild-type and mutant forms of p53, p28 offers a broad therapeutic approach, overcoming one of the key challenges in cancer treatment: the loss or mutation of p53 function.

Preclinical studies have consistently demonstrated the safety and efficacy of p28 in various cancer models. These findings have been supported by early-phase clinical trials, which have evaluated the peptide’s potential in treating a range of solid tumors, including glioblastoma and central nervous system tumors (NCT06102525; NCT01975116), as well as hepatocellular carcinoma (NCT05359861). Additionally, a Phase I trial (NCT00914914) further validated the safety profile of p28, reinforcing its therapeutic promise across diverse tumor types [78].

The unique properties of p28 as both a CPP and an anticancer agent set it apart from many other peptides under investigation. Its ability to penetrate cancer cells while simultaneously modulating key regulatory proteins, such as p53, positions p28 as a highly versatile therapeutic tool. Furthermore, the peptide’s amphipathic α-helical structure plays a critical role in its ability to interact with and traverse cellular membranes, enabling it to reach intracellular targets that are otherwise difficult to access.

As p28 continues to progress through clinical development, its dual functionality is being explored for combination therapies as well. Given its non-toxic nature and broad targeting capabilities, p28 could potentially enhance the efficacy of existing chemotherapeutic agents by improving their delivery to tumor cells while concurrently modulating key oncogenic pathways. This approach could lead to more effective and less toxic cancer treatments, particularly for patients with tumors that exhibit p53 mutations or resistance to conventional therapies [79,80].

### 2.9. SAP and SAP(E): Precision Drug Delivery Platforms for Targeted Cancer Therapy with Minimal Toxicity

A remarkable example in the realm of intracellular delivery is SAP ((VRLPPP)_3_). Derived from the N-terminal domain of γ-zein, SAP exhibits unique properties that make it an efficient tool for drug delivery. The peptide adopts a polyproline II (PPII) helical structure in aqueous solutions, which contributes to its high solubility in water and its lack of cytotoxicity, even at elevated concentrations [81]. This makes SAP an ideal candidate for applications where high peptide concentrations are necessary without inducing cell toxicity.

A modified version of SAP, known as SAP(E) ((VELPPP)_3_), was created by substituting the arginine residue with a glutamate residue. SAP(E) retains the PPII helical structure but is the first anionic CPP, possessing a negative charge at physiological pH. The negatively charged nature of SAP(E) circumvents the need for the initial electrostatic interactions with the cell membrane typically required for CPP internalization. Instead, it is hypothesized that SAP(E) aggregates on the cell surface before internalizing through a mechanism that appears to be independent of clathrin and likely involves caveolin-mediated endocytosis [82,83] (see Figure 3). This alternative entry mechanism highlights SAP(E)’s unique potential to deliver therapeutic agents without relying on common endocytic pathways that often lead to endosomal sequestration (see Figure 2).

Chemical modifications to SAP(E) further enhance its functionality as a drug delivery system. For instance, the addition of an Ac-CGGW linker at the N-terminus of the primary structure allows for the conjugation of biologically active substances. This linker includes activated thiol and aminooxy functionality, enabling the formation of a stable oxime bond, which is critical for attaching drugs to the peptide. A significant example of this is the conjugation of the anticancer drug doxorubicin. By linking doxorubicin to SAP(E), the conjugate was able to efficiently cross cell membranes and deliver the drug into the cytoplasm [84]. Once inside the cell, the conjugate can be cleaved by glutathione, releasing the active drug into the nucleus, where it exerts its anticancer effects by targeting DNA intercalation and inhibiting topoisomerase II, crucial for preventing cancer cell proliferation [85].

The ability of SAP(E) to deliver doxorubicin effectively into cancer cells has been validated through studies conducted in MCF-7 and HT-29 cancer cell lines. These studies demonstrated that the SAP(E)-doxorubicin conjugate enhances cellular uptake and significantly increases cytotoxicity compared with free doxorubicin. This selective and efficient delivery mechanism addresses one of the critical challenges in cancer treatment: ensuring that chemotherapeutic agents penetrate tumor cells without affecting healthy tissues, thereby minimizing systemic toxicity.

SAP and its modified counterpart SAP(E) offer a powerful platform for delivering a wide range of therapeutic molecules. Their ability to bypass traditional endocytic pathways and deliver drugs directly to intracellular targets positions these peptides as promising candidates for cancer therapy. Moreover, the versatility of SAP’s chemical modifications allows for the conjugation of various drugs and biologically active molecules, enhancing the scope of its potential applications.

### 2.10. Bac1-24: A Multifunctional Platform for Targeted Macromolecular Therapies in Solid Tumors

One more proline-rich antimicrobial peptide that has shown significant promise in drug delivery and cancer therapy is Bac-7. Bac-7 belongs to the bactenecin family and features a sequence rich in proline, divided into five distinct structural regions: a charged cap (RRI), a degenerated repeat (RPRPPRLPRPRPRP), and three 14-residue repeats ((LPFPRPGPRPIPRP)_3_) [86]. Notably, shortened fragments of Bac-7, despite their simplified structure, retain the ability to penetrate cells without exhibiting membranolytic activity, making them efficient candidates for intracellular delivery.

The antimicrobial activity of Bac-7, specifically its cell-permeant properties, is primarily localized within the first 24 residues of the peptide (Bac1-24). This N-terminal region is amphipathic, with distinct charged and hydrophobic domains, which facilitate its ability to traverse cellular membranes while maintaining a separation between its polar and non-polar regions [87]. This structural arrangement is crucial for Bac-7’s function, as it allows for the efficient delivery of non-covalently bound proteins into cells, indicating its potential as a CPP for therapeutic applications.

Bac-7’s utility extends beyond its antimicrobial activity, as demonstrated in studies focusing on its potential to deliver therapeutic polypeptides. One such example is the development of a thermally responsive polypeptide inhibitor of c-Myc, a well-known oncogenic protein. In this study, Bac1-24 was conjugated to an elastin-like polypeptide (ELP) fused with the H1 peptide, which blocks c-Myc/Max dimerization. The Bac1-24-ELP-H1 conjugate efficiently localized to the nucleus in a subset of cells and exhibited superior inhibitory activity on MCF-7 breast cancer cell proliferation compared with other CPPs, such as Penetratin and Tat. This enhanced inhibitory effect was attributed to Bac1-24’s ability to deliver the c-Myc inhibitory polypeptide more effectively [88].

Further investigation of Bac-7’s potential as a drug delivery vehicle was carried out by Massodi et al., who developed a polypeptide carrier system using Bac1-24 as a CPP for targeted cancer therapy [89]. In this system, Bac1-24 was fused to an ELP at the N-terminus, while a p21-derived 23-amino acid peptide was attached at the C-terminus, creating Bac1-24-ELP1-p21. This conjugate exhibited both cytoplasmic and nuclear localization in SKOV-3 ovarian cancer cells, where it induced caspase activation, PARP cleavage, and cell cycle arrest in the S and G2/M phases. This multi-pronged inhibitory effect underscores Bac1-24’s utility in targeted cancer treatment, particularly for solid tumors [89].

The versatility of Bac-7 and its shortened derivatives, such as Bac1-24, lies in their ability to act as delivery vehicles for therapeutic macromolecules, particularly polypeptides. Their amphipathic structure allows for efficient cellular uptake without causing membrane disruption, while their ability to localize to both the cytoplasm and nucleus expands their range of applications. The studies on Bac1-24-ELP1 and Bac1-24-ELP-H1 demonstrate its significant potential in cancer therapy, particularly for tumors that rely on dysregulated cell cycle proteins, such as c-Myc.

Overall, Bac-7 and its derivatives provide a robust platform for developing novel macromolecular therapies targeting solid tumors. Their ability to deliver functional proteins and peptides directly into cancer cells, coupled with their non-toxic profile, places them as promising candidates for further exploration in cancer therapeutics.

### 2.11. BIM-SAHB_A_ and SAHB_D_: Overcoming Apoptosis Resistance in Cancer via Targeted BH3- and MCL-1 Inhibition

Researchers have also explored strategies to restore apoptosis in cancer cells that have developed resistance to cell death mechanisms. A key approach has focused on targeting the BH3-only proteins, particularly BIM, a pro-apoptotic mediator within the BCL-2 family. BIM interacts directly with anti-apoptotic proteins like BCL-2, possessing one of the most potent BH3 death domains among its family members. This interaction is critical for regulating apoptosis, and its manipulation offers a promising strategy for overcoming apoptosis resistance in hematological malignancies.

One notable example of this approach is the work conducted by LaBelle and colleagues [90], who developed BIM-SAHB_*A*_, a stapled BIM BH3 helix designed to restore BH3-dependent cell death in resistant cancer cells. BIM-SAHB_*A*_ contains an i, i + 4 all-hydrocarbon crosslink between positions 154 and 158 of the BH3 domain, which stabilizes the peptide in an α-helical conformation, enhancing its binding affinity and functional stability. This stapling strategy enables BIM-SAHB_*A*_ to reactivate apoptosis both in vitro and in vivo by specifically mimicking the natural BH3 death domain [90].

Mechanically, BIM-SAHB_*A*_ functions by blocking the anti-apoptotic sequestration of BAX/BAK BH3 helices, crucial proteins in the intrinsic apoptosis pathway. By doing so, it facilitates the release of mitochondrial cytochrome c, a key event in apoptosis induction, in a BAX/BAK-dependent manner. This release ultimately activates caspase-3/7, driving programmed cell death in resistant hematologic cancer cells. The ability of BIM-SAHB_*A*_ to restore apoptosis in cells that have otherwise evaded cell death underscores its therapeutic potential, particularly in cancers where standard treatments have failed to induce sufficient apoptotic responses [90].

A similarly important example is the targeting of MCL-1, an anti-apoptotic member of the BCL-2 family that often mediates chemoresistance in various cancers. In this context, researchers developed a peptide known as SAHB_*D*_ (EDIIRNIAR(R5)LAQVGD(S8)NLDRSIW), which forms a complex with native MCL-1, effectively disrupting its interactions with pro-apoptotic proteins. MCL-1’s primary function is to neutralize proteins like BAX and BAK, preventing the initiation of apoptosis. However, when MCL-1 is bound by SAHB_*D*_, its anti-apoptotic activity is inhibited, thereby sensitizing the cancer cells to apoptosis triggered by death receptor stimulation [91] (see Figure 1).

The strategic inhibition of MCL-1 using peptides like SAHB_*D*_ has been shown to promote caspase-dependent apoptosis, particularly in cancer cells that rely on MCL-1 for survival. This interaction opens the door for new therapeutic models designed to specifically reactivate apoptosis in chemoresistant tumors, where pathological cell survival is driven by overexpression or dysregulation of MCL-1.

These findings demonstrate the therapeutic potential of targeting BH3-only proteins and the BCL-2 family in cancer therapy. By leveraging peptides that can disrupt the interactions between pro- and anti-apoptotic proteins, researchers are developing innovative strategies to reactivate apoptosis in resistant cancers. The BIM-SAHB_*A*_ and SAHB_*D*_ models highlight the broader application of stapled peptides and protein interaction inhibitors in overcoming apoptosis resistance, a significant hurdle in the treatment of hematologic malignancies and solid tumors [92,93].

### 2.12. ALRN-6924: A Stapled Peptide Restoring p53 Function for Targeted Cancer Therapy

Another promising therapeutic in cancer treatment is ALRN-6924 (Sulanemadlin), a stapled peptide designed to target the p53 pathway. ALRN-6924 is engineered to mimic the N-terminal domain of the p53 tumor suppressor protein, a critical regulator of apoptosis and cell cycle control. This peptide exhibits high-affinity binding to MDM2 and MDMX (also known as MDM4), which are negative regulators of p53. By inhibiting these proteins, ALRN-6924 activates p53 signaling, leading to tumor suppression in cancers with wild-type TP53 [94].

The ability of ALRN-6924 to block MDM2 and MDMX, thereby restoring p53 activity, offers a potent therapeutic strategy, particularly for cancers that retain functional p53 but are suppressed by overactive MDM2/MDMX interactions. In wild-type TP53 (TP53-WT) cells, p53 reactivation by ALRN-6924 leads to growth inhibition and induction of apoptosis. Notably, the peptide has shown synergistic effects when combined with anti-PD-1 immunotherapy, enhancing immune cell infiltration and improving overall survival in preclinical models [94].

The structure of ALRN-6924 has been iteratively optimized through structure-activity relationship (SAR) studies to improve its solubility, cell permeability, pharmacokinetics, and safety profile. Once internalized, ALRN-6924 undergoes proteolysis, producing a long-acting metabolite that retains strong binding to MDM2 and MDMX with slow dissociation kinetics. This slow-release mechanism prolongs the peptide’s effects, resulting in sustained p53 activation. At therapeutic doses, ALRN-6924 induces cell cycle arrest and apoptosis in TP53-WT tumors. At lower doses, the peptide transiently halts the cell cycle in normal, healthy tissues, offering chemoprotection without impacting TP53-mutant cancer cells [95].

ALRN-6924’s mechanism of action has been shown to trigger a unique pharmacodynamic response by upregulating the transcription factor p53. This response is evidenced by increased serum levels of MIC-1, a protein regulated by p53, which remains elevated for more than 48 h after administration, despite the peptide’s relatively short plasma half-life of 5.4 h. These findings underscore the peptide’s ability to achieve prolonged therapeutic effects even after a single dose [96].

The anticancer potential of ALRN-6924 has been explored across various cancer types. In preclinical studies involving estrogen receptor-positive (ER+) breast cancer, the peptide demonstrated synergistic effects when combined with chemotherapeutic agents such as paclitaxel and eribulin. Notably, ALRN-6924 significantly increased the apoptotic rate in TP53-WT breast cancer cells, while no such effect was observed in TP53-mutant cells, highlighting its specificity for p53-driven pathways. These results suggest that ALRN-6924, in combination with standard chemotherapy, could enhance therapeutic efficacy in patients with ER+ breast cancer [97].

In addition to breast cancer, ALRN-6924 has shown promise in hematological malignancies. In a preclinical model of acute myeloid leukemia (AML), treatment with ALRN-6924 led to a threefold increase in median survival in mice transplanted with human leukemia cells. These findings were particularly encouraging, as they demonstrated the potential for significant survival benefits in a challenging cancer type where current therapies often fall short [98].

ALRN-6924’s broad applicability is further supported by multiple ongoing clinical trials. These trials are investigating its use both as a monotherapy and in combination with other treatments for a variety of cancers, including AML, solid tumors, and hormone receptor-positive breast cancers. Trials such as NCT02264613, NCT04022876, NCT03654716, and NCT05622058 aim to further elucidate the peptide’s clinical efficacy, optimal dosing regimens, and potential for integration into standard cancer therapies.

As clinical trials progress, ALRN-6924 could arise as a key player in the development of precision cancer therapies that leverage the tumor-suppressive power of p53. Its ability to modulate p53 activity in cancer cells that retain TP53-WT, together with its chemoprotective effects in normal tissues, makes ALRN-6924 a valuable candidate for both solid and hematological malignancies.

### 2.13. P1pal-7: A Versatile Pepducin Targeting PAR1 for Cancer Therapies

Another innovative class of therapeutic peptides that is attracting attention is pepducins. Among these, P1pal-7 (also known as PZ-128) has emerged as a potent 7-mer palmitoylated pepducin (palmitate-KKSRALF) with applications in oncological therapies. P1pal-7 acts as a reversible inhibitor of protease-activated receptor 1 (PAR1), a G protein-coupled receptor (GPCR) present on platelets and vascular cells. This inhibition occurs by targeting the intracellular surface of PAR1, effectively disrupting the signal transduction processes that are critical for platelet activation and other downstream cellular responses [99,100] (see Figure 1).

P1pal-7 functions by mimicking the off-state conformation of the juxtamembrane region of PAR1’s third intracellular loop, thereby blocking its interaction with intracellular G proteins. This unique mode of action, which interferes with signaling at the cytoplasmic surface of the receptor, distinguishes pepducins from traditional receptor inhibitors that target extracellular domains [101]. The ability of P1pal-7 to inhibit PAR1 activation has been validated in vivo [102].

Given its ability to target the intracellular pathways of PAR1, it has been explored in oncology for its potential to inhibit cancer progression [103]. In preclinical cancer studies, nude mice were inoculated with the invasive breast cancer cell line MCF7-PAR1/N55. Mice treated with P1pal-7 exhibited a 62% reduction in tumor growth compared with controls, highlighting the peptide’s efficacy in slowing cancer progression [104]. Additionally, P1pal-7 treatment significantly decreased tumor blood vessel density by 75%, underscoring its ability to inhibit angiogenesis—a critical factor in tumor growth and metastasis. These results suggest that P1pal-7’s capacity to interfere with PAR1 signaling may offer therapeutic benefits in cancer by targeting both tumor cells and the tumor microenvironment.

Pepducins, as a broader class, represent an exciting therapeutic platform due to their unique ability to modulate GPCR signaling from within the cell. Unlike traditional small molecules or antibodies that block receptor activity from the extracellular side, pepducins disrupt intracellular signaling cascades by binding to the cytoplasmic domains of GPCRs [105]. This mechanism allows for more specific modulation of receptor activity, offering a novel approach to receptor inhibition with favorable pharmacodynamic and pharmacokinetic properties. Furthermore, pepducins are relatively simple to synthesize and can be produced in large quantities, making them attractive candidates for drug development [106].

Preclinical testing of pepducins has revealed low toxicity profiles, suitable biodistribution, and significant therapeutic potential in multiple disease models, including cancer, cardiovascular diseases, and systemic inflammation. These characteristics, combined with their ease of synthesis, position pepducins as versatile therapeutic agents that could be applied to a broad range of clinical conditions [107,108].

### 2.14. EN1-iPeps: Homeodomain-Derived CPPs Targeting Oncogenic Transcription Factors for Selective Cancer Therapy

Another cutting-edge therapeutic approach involves the use of homeodomain-derived CPPs. A homeodomain is a 60 amino acid DNA-binding domain present in homeoproteins, encoded by a 180 bp homeobox sequence. Its structure consists of three α-helices, with two forming a helix-turn-helix (HTH) motif that interacts with the DNA minor groove, while the third, the “recognition helix”, engages with the DNA major groove [109,110]. These peptides have been specifically designed to block the function of transcription factors implicated in the progression of aggressive cancers. A prime example is the neural-specific transcription factor Engrailed 1 (EN1), which is overexpressed in particularly aggressive cancers. Notably, EN1 has been shown to play a critical role in promoting cancer cell survival and resistance to treatment.

Experimental studies have demonstrated that knockdown of EN1 triggers potent and selective apoptosis in cancer cells, while overexpression of EN1 in normal cells leads to the activation of survival pathways, thereby conferring resistance to standard chemotherapeutic agents. These findings underscore the potential of targeting EN1 as a therapeutic strategy for inhibiting cancer progression, particularly in cancers where EN1 is abnormally expressed [111].

To inhibit the function of EN1, researchers have developed synthetic interfering peptides (iPeps). These iPeps are engineered to include EN1-specific sequences that are essential for mediating the transcription factor’s protein-protein interactions.The EN1-iPep, consisting of 29 residues, including the CPP, is derived from the EN1 transcription factor and contains a conserved hexapeptide motif (WPAWVY) common to the homeodomain superfamily. Its N-terminus is engineered with the CPP/NLS sequence KKKRV from the Simian Virus 40 (SV40) large T-antigen, facilitating its internalization across plasma and nuclear membranes [112]. These EN1-iPeps have been shown to elicit a strong apoptotic response in tumor cells that overexpress EN1, effectively halting cancer cell survival without causing toxicity to normal cells that do not express EN1 [113].

The selective nature of these peptides is a critical advantage, as they specifically target cancer cells with high EN1 expression while sparing normal cells. This precision not only enhances therapeutic efficacy but also reduces the risk of off-target effects, which is a significant concern in many traditional cancer therapies. Moreover, EN1-iPeps have been found to inhibit EN1’s role in activating intrinsic inflammatory pathways associated with tumor survival, which is particularly relevant in basal breast cancer, a subtype of breast cancer known for its aggressive behavior and poor prognosis [111] (see Figure 1).

The engineering of homeoprotein-derived CPPs, such as EN1-iPeps, represents a novel and selective therapeutic strategy. By directly inhibiting transcription factors that drive cancer progression, these peptides offer a promising alternative to traditional approaches that target downstream effects rather than the root cause of transcriptional dysregulation. The ability to design iPeps that interfere with specific protein-protein interactions necessary for the function of oncogenic transcription factors opens new avenues for targeted cancer therapy.

### 2.15. Vectocell^®^/DPVs: Innovative Peptide Vectors for Targeted Drug Delivery and Combatting Multidrug Resistance in Cancer

A further promising class of CPPs, known as Vectocell^®^ or Diatos Peptide Vectors (DPVs), has been identified for their potential in drug delivery. These cationic CPPs are derived from human heparin-binding proteins and anti-DNA antibodies, including well-characterized proteins such as superoxide dismutase (DPV3 and DPV3/10), platelet-derived growth factor (DPV6), epidermal-like growth factors (DPV7-DPV7b), intestinal mucin (DPV10/6), apolipoprotein B (DPV1047), and the cationic antimicrobial protein of 37 kDa (CAP 37) [114].

These peptides are highly versatile and can facilitate the internalization of a broad range of molecules, from small compounds of a few Daltons (Da) to large macromolecules up to 200 kDa. This flexibility in molecular size makes DPVs highly applicable for a variety of therapeutic payloads. The internalization of DPVs, except for DPV1047, relies on interactions with glycosaminoglycans (GAGs) present on the cell surface. The uptake mechanism is primarily mediated by active caveolar endocytosis, a pathway closely associated with signal transduction and intracellular trafficking through lipid raft-associated molecules. This mechanism not only enhances the internalization of the conjugated therapeutic agents but also allows for the specific delivery of molecules to targeted subcellular locations, including the cytoplasm or nucleus depending on the peptide utilized.

The potential of DPVs has been further demonstrated in studies aimed at improving the therapeutic index of doxorubicin, a widely used chemotherapeutic agent. In these studies, doxorubicin was chemically conjugated to short peptide sequences (15 to 23 amino acids) from the Vectocell family, using different linkers to optimize drug delivery. One particularly successful strategy involved conjugating doxorubicin to DPV1047 using an ester linker at the C14 position of the drug. This conjugated form of doxorubicin exhibited significantly improved efficacy in colon and breast tumor models, offering better tumor suppression while reducing toxicity to normal cells, a critical advantage in minimizing off-target effects [115].

Moreover, the conjugation of doxorubicin with DPV1047 demonstrated enhanced antitumor activity in a model of doxorubicin-resistant cancer, suggesting that this approach may be effective in overcoming multidrug resistance (MDR). MDR is a major hurdle in cancer treatment, where cancer cells develop resistance to multiple chemotherapeutic agents, leading to poor clinical outcomes. The ability of the DPV-doxorubicin conjugate to bypass these resistance mechanisms holds significant potential for enhancing the efficacy of existing chemotherapies, particularly in treatment-resistant cancers.

The importance of this finding cannot be overstated, as MDR continues to pose a significant challenge in oncology, with many cancers becoming refractory to standard treatments. The DPV1047-doxorubicin conjugate represents a promising solution by not only increasing the delivery of active drug to tumor cells but also reducing systemic toxicity—a critical goal in cancer therapy.

Overall, Vectocell^®^/DPVs offer a highly adaptable platform for the targeted delivery of therapeutic agents, particularly in cancer treatment. Their ability to enhance drug internalization, minimize toxicity, and overcome resistance mechanisms such as MDR underscores their potential as an innovative tool in the development of more effective cancer therapies.

### 2.16. CPPecp: Targeting Tumor Cell Migration and Angiogenesis for Comprehensive Cancer Therapy

Another significant advancement in the field of CPPs is the identification of heparan sulfate-binding (HS-binding) CPPs. These peptides, which target heparan sulfate (HS) on the surface of cancer cells, have shown great promise in modulating tumor cell behavior. A study conducted by Chen C. J. et al. investigated a series of such CPPs derived from natural proteins, including CPPecp (NYRWRCKNQN), which was identified from a critical HS-binding region in hRNase3, a member of the ribonuclease family known for its in vitro antitumor activity [116].

CPPecp demonstrated a unique set of functions that enhance its therapeutic potential. It exhibited strong binding affinity to the surfaces of tumor cells, particularly those expressing elevated levels of HS, a GAG involved in cell signaling and the regulation of cancer cell behavior. This selective binding is crucial for reducing off-target effects and ensuring that therapeutic agents are concentrated in the tumor microenvironment, thereby increasing efficacy.

In addition to its robust binding capabilities, CPPecp has shown significant inhibitory effects on cancer cell migration. Cell migration is a key process in cancer metastasis, and the ability to disrupt this process could have profound implications for controlling cancer spread. In vitro studies demonstrated that CPPecp effectively hampers the motility of cancer cells, suggesting its potential as an anti-metastatic agent. This inhibition of migration can disrupt the dissemination of cancer cells to secondary sites, which is often a leading cause of cancer-related mortality.

Another critical function of CPPecp is its capacity to suppress angiogenesis, both in vitro and in vivo. Angiogenesis, the formation of new blood vessels, is a hallmark of tumor growth, as it provides cancer cells with the necessary oxygen and nutrients to proliferate. By inhibiting angiogenesis, CPPecp targets the tumor’s ability to sustain itself, thereby limiting tumor growth and progression. The potential to interfere with angiogenic processes positions CPPecp as a potential anti-angiogenic agent, offering a dual mechanism of action—both inhibiting tumor cell migration and cutting off the tumor’s vascular supply.

These findings highlight the multifunctionality of CPPecp as a therapeutic agent with the ability to modulate several critical pathways involved in tumor progression. Its dual action in suppressing both migration and angiogenesis makes it a promising candidate for further development in cancer therapy, particularly in the context of metastatic cancers, where targeting both primary tumor growth and secondary spread is essential [116].

### 2.17. Melittin and Its Derivatives: Harnessing Venom-Derived Peptides for Targeted Cancer Therapy and Drug Delivery

On the other hand, an intriguing candidate in the realm of CPPs with significant therapeutic potential is melittin, a cationic peptide derived from European honeybee, *Apis mellifera*, venom. Melittin, with the sequence GIGAVLKVLTTGLPALISWIKRKRQQ, adopts an amphipathic α-helix structure, a characteristic that allows it to interact efficiently with lipid membranes. At low concentrations, melittin integrates into neutral lipid bilayers and phospholipid membranes, while at higher concentrations, it forms transmembrane pores, which contribute to its membrane-disrupting and cytolytic properties [117,118].

Melittin’s ability to disrupt cellular membranes underpins its broad-spectrum anti-infective and anticancer activities. It has demonstrated potent cytotoxic effects against a wide range of tumor cells, which makes it an attractive candidate for therapeutic development. However, the same properties that make melittin effective can also pose challenges, as its non-selective membrane-disrupting capabilities may lead to off-target toxicity. Consequently, a number of melittin derivatives and analogs have been developed to enhance druggability and improve its selectivity for tumor cells [119,120].

One promising approach to improving melittin’s specificity involves the design of chimeric peptides that combine melittin with other functional domains. For instance, combining melittin with a pro-apoptotic peptide has shown efficacy in selectively targeting tumor-associated macrophages (TAMs), a key component of the tumor microenvironment that supports cancer progression. By inducing apoptosis in TAMs, this chimeric peptide was able to reduce tumor growth, highlighting its potential for targeting both tumor cells and the surrounding supportive stroma [121,122].

Furthermore, modified melittin-derived peptides containing arginine (Arg) and histidine (His) residues have demonstrated enhanced efficacy in cancer therapy by inhibiting both cell proliferation and metastasis. These modifications not only improve the peptide’s ability to target tumor cells more specifically but also enable it to efficiently deliver therapeutic agents such as siRNAs into the cytoplasm. This functionality is particularly valuable in the context of gene therapy, where precise intracellular delivery of nucleic acids is crucial for therapeutic efficacy [123].

In addition to these modifications, truncated versions of melittin have been developed to enhance endosomal escape and improve transfection efficiency in eukaryotic cells. The truncated peptides retain melittin’s capacity for membrane penetration but with reduced cytotoxicity, making them safer for therapeutic applications. This is particularly relevant in drug delivery systems, where endosomal entrapment of therapeutic agents is a significant barrier to effective intracellular delivery.

Moreover, the N-terminal fragment of melittin has shown high efficacy in facilitating the cellular uptake of nanocrystals and large proteins. This fragment enhances the delivery of these macromolecules into the cytoplasm, where they can exert their therapeutic effects, further demonstrating melittin’s versatility in drug delivery applications.

These findings suggest that the cytolytic properties of venom-derived peptides like melittin can be harnessed and refined into safe and effective therapeutics for various biomedical applications. By modulating the peptide’s structure and combining it with other therapeutic domains, researchers have expanded its potential beyond its native cytotoxic activity, allowing it to serve as a targeted therapeutic tool in cancer treatment, antimicrobial therapy, and drug delivery systems.

### 2.18. Lycosin-I and R-Lycosin-I: Optimizing Venom-Derived Peptides for Enhanced Anticancer Efficacy

Another notable peptide with significant potential in cancer therapy is Lycosin-I. Lycosin-I (RKGWFKAMKSIAKFIAKEKLKEHL) is a linear cationic peptide derived from the venom of the *Lycosa singorensis* spider, exhibiting potent anticancer properties through its ability to activate the mitochondrial death pathway, leading to apoptosis in tumor cells. One of its mechanisms involves the upregulation of p27, a cyclin-dependent kinase inhibitor that plays a crucial role in inhibiting cell proliferation [124]. When interacting with lipid membranes, Lycosin-I adopts an amphiphilic α-helix structure, facilitating its integration into cellular membranes and triggering apoptosis [125].

Studies have shown that Lycosin-I can significantly inhibit tumor growth across various cancer models [126]. Of particular interest is its ability to induce apoptosis and impede the migration of prostate cancer cells, a key factor in the prevention of metastasis [127]. Upon entering the cytoplasm, Lycosin-I initiates intracellular signaling pathways that reduce both cell proliferation and induce cell death, making it a strong candidate for further development as an anticancer agent.

Despite these promising properties, the potential of Lycosin-I as a therapeutic agent has been constrained by challenges in cell penetration and its limited effectiveness in targeting solid tumors. These barriers have spurred efforts to develop improved variants of the peptide. One significant modification involves replacing the original lysine residues in Lycosin-I with arginine, resulting in a new variant known as R-Lycosin-I (RGWFRAMRSIARFIARERLREHL). The introduction of arginine was intended to enhance the peptide’s binding affinity to cell membranes as well as its overall bioavailability—both critical factors in improving its anticancer efficacy [128].

R-Lycosin-I has demonstrated greater anticancer activity compared with the original Lycosin-I, particularly in terms of its ability to penetrate solid tumor cells. The variant exhibited improved membrane permeability, allowing for more efficient entry into cancer cells, where it could exert its cytotoxic effects. Notably, R-Lycosin-I’s modified structure showed better physicochemical properties, including altered secondary structure, hydrodynamic size, and zeta potential, all of which contributed to its enhanced performance in cellular environments [128].

The improved bioavailability and membrane-binding properties of R-Lycosin-I represent a significant advancement in the development of spider venom-derived peptides for cancer treatment. By overcoming some of the limitations associated with Lycosin-I, particularly in the context of targeting solid tumors, this variant offers new avenues for therapeutic intervention. The structural modifications not only enhance the peptide’s anticancer activity but also improve its stability and efficacy, potentially broadening its applicability across different cancer types.

### 2.19. Pardaxins: Amphipathic Peptides Targeting Membrane Disruption and Mitochondrial Dysfunction in Cancer Therapy

A distinct class of promising therapeutic peptides is pardaxins. Pardaxins (P1 to P5) are cytotoxic peptides secreted by Moses sole fishes of the genus *Pardachirus* (*P. marmoratus* and *P. pavoninus*) into seawater. These peptides share a common structure: a 33-amino acid single-chain acidic peptide, rich in ASP, SER, GLY, and ALA, while lacking ARG, TYR, and TRP. Structurally, pardaxins feature an N-terminal hydrophobic α-helix connected to a C-terminal amphiphilic α-helix via a dipeptide linker (SerPro) [129,130,131]. Their amphipathic nature allows pardaxins to insert into biological membranes, where they create pores, resulting in cytolysis [132,133].

Pardaxins’ hydrophobic and pore-forming characteristics enable them to disrupt both healthy and tumor cell membranes, leading to cell lysis. Studies have demonstrated that pardaxin exhibits antitumor activity against various cancer cell lines, including human fibrosarcoma (HT-1080) and epithelial carcinoma (HeLa) cells. Importantly, pardaxin shows selectivity, as it does not induce lysis in human red blood cells at concentrations up to 15 μg/mL. Further research revealed that pardaxin dose-dependently inhibits the proliferation of HT1080 cells and triggers programmed cell death in HeLa cells [134,135,136]. These findings underscore its potential as an anticancer agent with selective cytotoxicity towards cancer cells.

Additionally, pardaxin has demonstrated potential in leukemia treatment. Uen et al. [137] showed that pardaxin induces the differentiation of leukemic cells into macrophage-like cells with immunostimulatory functions, such as enhanced phagocytosis and superoxide anion production. In leukemic cell lines THP-1 and U937, pardaxin significantly reduced cell viability and induced cell cycle arrest at the G0/G1 phase, further highlighting its ability to modulate immune functions and inhibit leukemic cell proliferation. These preliminary findings suggest pardaxin as a promising candidate for leukemia therapy, though further studies are necessary to explore its full therapeutic potential in this context.

In addition to leukemia, pardaxin has been evaluated for its anticancer activity in ovarian cancer cells, particularly PA-1 (teratocarcinoma) and SKOV3 (adenocarcinoma). In these models, pardaxin activates a cytotoxic mechanism that involves the overproduction of reactive oxygen species (ROS) in mitochondria. This ROS accumulation leads to mitochondrial membrane depolarization, which disrupts mitochondrial membrane potential, subsequently activating pro-caspases 9 and 3. The mitochondrial apoptosis pathway is further supported by the upregulation of t-Bid and Bax, key regulators of cell death. Ultimately, pardaxin induces mitochondria-mediated apoptosis in ovarian cancer through excessive mitophagy and ROS generation [138]. These findings illustrate pardaxin’s capacity to target mitochondrial function, offering a pathway-specific approach to anticancer therapy.

Moreover, pardaxin has shown anticancer activity in oral squamous cell carcinoma (OSCC). In in vitro assays using OSCC cell lines (SCC-4), pardaxin reduced cell viability in a dose-dependent manner. Caspase-3 activation assays revealed that pardaxin treatment significantly increased cleaved caspase-3 expression, indicative of apoptosis induction. Cell cycle analysis further demonstrated that pardaxin arrests cells in the G2/M phase, thereby inhibiting proliferation in SCC-4 cells. In a DMBA-induced hamster buccal pouch model, pardaxin treatment significantly reduced prostaglandin E2 (PGE2) levels and mitigated carcinogenesis, suggesting its potential as an adjuvant chemotherapy agent for OSCC and oral cancer [139].

In conclusion, pardaxins offer novel mechanisms to combat resistant cancers [140,141]. Due to their ability to selectively disrupt cancer cell membranes, modulate mitochondrial function, and inhibit cell proliferation, pardaxins are promising candidates for further development in new anticancer therapies.

### 2.20. BT1718: A Bicyclic Peptide Toxin Conjugate Targeting MT1-MMP for Precision Cancer Therapy

Bicycle Toxin Conjugates (BTCs) represent a novel class of therapeutics that utilize bicyclic peptide technology for targeted drug delivery. BT1718 is a prime example, designed to selectively target the hemopexin domain of membrane type 1 matrix metalloproteinase (MT1-MMP), a protein overexpressed in many solid tumors and associated with poor prognosis due to its critical role in cell invasion and metastasis [142,143].

The therapeutic mechanism of BT1718 revolves around its bicyclic peptide component, which binds with high affinity to MT1-MMP, thereby exploiting its overexpression on tumor cells. Once bound, BT1718 delivers its cytotoxic payload, DM1 (N2′-deacetyl-N2′-[3-mercapto-1-oxopropyl]-maytansine), a potent tubulin inhibitor. The DM1 is linked to the peptide via a cleavable disulfide linker that is specifically designed to be cleaved within the tumor microenvironment, ensuring that the active cytotoxic agent is released precisely at the tumor site [144,145]. This highly targeted approach minimizes off-target effects, which are a significant concern with many conventional therapies.

Upon release, DM1 binds to microtubules, disrupting the cellular machinery necessary for tumor cell division, leading to cell death and a consequent reduction in tumor size. This precision-targeting mechanism distinguishes BT1718 from traditional MMP inhibitors, which aim to block the activity of the enzyme itself. Instead, BT1718 capitalizes on the presence of MT1-MMP as a delivery vehicle, allowing for selective toxicity in cancer cells while sparing normal tissue [142].

Preclinical studies have shown that BT1718 demonstrates significant efficacy against treatment-resistant cancers, particularly those where conventional therapies have failed. Additionally, BT1718 has displayed reduced toxicity compared with other potent cancer treatments, a feature that underscores its potential as a safer and more effective therapeutic option for aggressive cancers. By specifically targeting MT1-MMP, BT1718 effectively induces tumor cell death while minimizing systemic toxicity, which is a considerable advantage in oncology, where the balance between efficacy and safety is paramount.

The development of BT1718 has reached a critical milestone with ongoing clinical trials (NCT03486730), aiming to validate its efficacy and safety in human patients. The promising preclinical data and its favorable safety profile in early-phase studies position BT1718 as a highly innovative therapeutic candidate. Its potential to treat aggressive and treatment-resistant cancers could significantly impact the landscape of cancer therapy, offering new hope for patients with limited treatment options.

### 2.21. ^177^Lu-DOTA^0^-Tyr^3^-Octreotate: A Game-Changer in Peptide Receptor Radionuclide Therapy for Neuroendocrine Tumors

A significant advancement in targeted cancer therapies is ^177^Lu-DOTA^0^-Tyr^3^-Octreotate. This radioconjugate is part of a cutting-edge approach known as Peptide Receptor Radionuclide Therapy (PRRT) and combines the somatostatin analog Tyr^3^-octreotate (TATE) with the chelating agent DOTA, radiolabeled with the β-emitting isotope lutetium-177 (^177^Lu). This dual-purpose conjugate serves both imaging and therapeutic roles, as it binds with high affinity to type 2 somatostatin receptors (SSTR2), which are overexpressed in neuroendocrine tumor (NET) cells.

Upon binding to SSTR2-positive cells, the radioconjugate is internalized, delivering a cytotoxic dose of β radiation directly to the targeted tumor cells. This selective delivery allows for localized cell destruction, minimizing damage to surrounding healthy tissue and enhancing the precision of the therapy. The highly targeted nature of ^177^Lu-DOTA^0^-Tyr^3^-Octreotate positions it as a major advancement in the treatment of NETs [146].

One of the key advantages of this therapy is its superior tumor uptake and extended residence time within the tumor microenvironment, which allows for sustained cytotoxic effects. Despite its prolonged retention in tumors, ^177^Lu-DOTA^0^-Tyr^3^-Octreotate exhibits reduced whole-body retention, thereby mitigating the risk of bone marrow toxicity and preserving renal function—a common concern with radiotherapy. To further enhance patient safety, renal protective agents are often used during treatment, significantly reducing the risk of nephrotoxicity. In fact, patients generally experience mild adverse effects, and the therapeutic response often extends beyond two years, underscoring the long-term benefits of this radioconjugate [147].

The radioligand properties of ^177^Lu-DOTA^0^-Tyr^3^-Octreotate also facilitate real-time monitoring of treatment efficacy, allowing for precise dose adjustments based on imaging data. This adaptability in therapy planning further enhances its clinical utility, offering oncologists a robust tool for both diagnosis and treatment [148,149].

Clinical trials have confirmed the efficacy of ^177^Lu-DOTA^0^-Tyr^3^-Octreotate across a range of neuroendocrine tumors, including pulmonary NETs (NCT03325816) [150], bronchial and gastroenteropancreatic NETs [151], and progressive NETs [152]. Approved by the FDA as Lutathera® in January 2018 and by the EMA in September 2017, it is the first radiopharmaceutical agent approved for the treatment of unresectable or metastatic, progressive, well-differentiated (G1 and G2) SSTR-positive gastroenteropancreatic NETs (GEP-NETs) in adults [153].

This approval marked a significant milestone in cancer treatment, offering patients with advanced neuroendocrine tumors an effective and targeted therapeutic option. The success of ^177^Lu-DOTA^0^-Tyr^3^-Octreotate exemplifies the potential of radioconjugates in modern oncology, combining precise tumor targeting with a strong therapeutic index. Its ability to deliver potent radiation directly to cancer cells while minimizing systemic side effects makes it a cornerstone of PRRT.

The development of novel CPPs, such as homeodomain-derived peptides, venom-derived peptides like melittin, and radioconjugates such as ^177^Lu-DOTA^0^-Tyr^3^-Octreotate, showcases the ability of these peptides to adapt to different therapeutic needs, whether by disrupting cancer cell membranes, modulating intracellular pathways, or delivering radiolabeled agents for both imaging and treatment. Furthermore, innovative approaches such as BTCs and modifications of classical CPPs highlight the capacity to refine their structure and function, thereby enhancing their specificity, stability, and efficacy.

As ongoing research continues to refine CPP technology, their integration into cancer treatment protocols offers a promising path toward more effective, personalized, and less toxic therapeutic strategies. The ability to selectively target aggressive and treatment-resistant cancers underscores their potential to expand the oncologist’s toolkit, providing new avenues to tackle some of the most challenging malignancies. Ultimately, CPPs are poised to play a central role in the future of cancer therapies, particularly in the development of precision medicine.

## 3. Overview of Cell Translocation Mechanisms of CPPs

CPPs utilize various mechanisms to cross cell membranes. They can enter through energy-dependent pathways, such as endocytosis, or through energy-independent routes, like membrane destabilization, via direct interactions with cellular membrane components [7]. Due to the diversity of these mechanisms, there is no single, universal experimental approach for characterizing the uptake and efficiency of CPPs. Instead, the choice of method depends on the specific nature of each CPP and its uptake process [154].

While many CPPs share common features, such as their cationic charge, their internalization pathways can vary across peptide families and are influenced by experimental conditions [10]. The intrinsic physicochemical properties of CPPs—such as charge, length, and structure—play a key role in determining the type of internalization. However, external factors, including the specific composition of the cell membrane, environmental pH, the nature of any attached cargo, and the concentration of CPPs, can also impact internalization pathways [155]. Notably, most CPPs utilize two or more internalization mechanisms, depending on the specific experimental conditions [10].

A key factor influencing CPP uptake is the landscape of secondary structures adopted by these peptides. Many CPPs, or specific segments of them, are intrinsically disordered under bulk conditions, meaning they lack a predominant set of meta-stable conformational states when observed in isolation (just solvated) [156]. However, in proximity to the cell membrane or interacting with proteins, a specific CPP can adopt critical structures that facilitate cellular uptake [156]. This makes the relationship between a peptide’s amino acid sequence and its relevant conformational states, and between these conformations and the uptake mechanisms, both complex and intriguing [7].

Moreover, for CPPs with potential cancer-targeting applications, interactions with cell surface receptors or membrane components are crucial not only for facilitating or hindering CPP translocation but also for providing a selectivity filter between malignant and non-malignant cells. The overexpression of specific cellular receptors in tumors, as well as the abundance of negatively charged phospholipids, can enhance the selective penetration of CPPs like Pep-1, MAP, and SAP into cancer cells (see Table 1).

Finally, understanding the complex interplay between a peptide’s properties, its structural tendencies, its interactions with the membrane and its components, and the role of any attached cargo is crucial for advancing the development and application of CPPs in therapeutic and research settings. In the following subsections, the main mechanisms of CPP uptake are described, while Figure 3 provides a graphical representation of these mechanisms along with the known pathways for each CPP discussed in the previous section.

### 3.1. Direct Translocation

CPPs can directly translocate cell membranes via energy-independent mechanisms, first proposed when their uptake was observed at low temperatures [157]. This single-step process involves mechanisms like inverted micelle formation, pore formation, or the ‘carpet’ model [158]. Positively charged CPPs interact with negatively charged membrane components, facilitating entry by destabilizing the membrane [10,159].

#### 3.1.1. Inverted Micelle Formation

Direct translocation initiates with electrostatic interactions between CPPs and the cell membrane, disrupting lipid organization and inducing membrane curvatures that evolve into inverted micelles. These micelles encapsulate CPPs and their cargo within a hydrophilic environment, facilitating membrane crossing. Upon destabilization of the micelle structure, CPPs and their cargo are released into the cytoplasm [157,160]. This mechanism is particularly effective for transporting hydrophilic compounds conjugated to peptides, as the micelle’s interior preserves the integrity of these conjugates.

#### 3.1.2. Direct Translocation via Pore Formation

The pore formation model encompasses two mechanisms: the ‘barrel-stave’ and ‘toroidal’ models. In the ‘barrel-stave’ model, characteristic of amphipathic α-helical peptides, peptides assemble into bundles forming pores; hydrophilic sides face the pore lumen, while hydrophobic regions interact with the lipid bilayer. The ‘toroidal’ model involves peptides forming α-helices upon membrane interaction, inducing the lipid monolayer to bend inward and create hydrophilic channels composed of both phospholipid headgroups and peptides [161,162]. This hydrophilic environment facilitates the translocation of CPPs and their cargo across the membrane.

#### 3.1.3. Carpet Model

In the ‘carpet’ model, CPPs align parallel to the membrane surface, binding electrostatically to negatively charged phospholipids and forming a carpet-like structure with hydrophobic regions embedding into the lipid bilayer. At critical peptide concentrations, this destabilizes the membrane, creating transient openings for CPP penetration. Alternatively, the ‘membrane-thinning’ effect suggests that CPP-lipid interactions rearrange lipids laterally, reducing membrane thickness and facilitating peptide insertion [158]. Both models underscore the importance of electrostatic interactions and high peptide concentrations for successful internalization.

### 3.2. Endocytosis as a Pathway for the Cellular Uptake of CPPs

At low concentrations, CPPs—especially when conjugated to cargo—are primarily internalized via energy-dependent endocytosis, involving uptake and subsequent endosomal escape. Endocytosis includes pinocytosis, the universal uptake of fluids and solutes, which comprises mechanisms like macropinocytosis, clathrin-mediated endocytosis (CME), caveolae-mediated endocytosis (CvME), and clathrin- and caveolae-independent pathways. The choice of endocytic route depends on cell type, differentiation state, and the physicochemical properties of the CPPs involved, such as surface reactivity [163].

#### 3.2.1. Macropinocytosis

Macropinocytosis has emerged as a key pathway for CPP uptake—a rapid, receptor-independent form of endocytosis driven by lipid rafts [163]. Induced by growth factors or stimuli, it involves actin-driven membrane ruffling and membrane folding to form macropinosomes that engulf extracellular fluid and materials [164]. Unlike receptor-mediated endocytosis, it requires no ligand-receptor interactions but involves cytoskeletal rearrangements regulated by kinases (Src, PI3K) and GTPases (Rho, Ras families) [165]. CPPs exploit this route by inducing macropinocytosis through membrane interactions for efficient uptake.

#### 3.2.2. Clathrin-Mediated Endocytosis (CME)

Clathrin-mediated endocytosis (CME) is a receptor-dependent pathway crucial for CPP internalization, involving the formation of clathrin-coated pits on the plasma membrane [163]. Ligand binding initiates clathrin lattice assembly, leading to membrane invagination. Dynamin-mediated scission releases vesicles that shed clathrin coats and fuse with early endosomes; maturation into late endosomes facilitates cargo release under acidic conditions [166]. CME encompasses five stages—initiation, cargo loading, membrane bending, vesicle scission, and coat disassembly—coordinated for efficient CPP uptake [160].

#### 3.2.3. Caveolae-Mediated Endocytosis (CvME)

Caveolae are flask-shaped plasma membrane invaginations abundant in endothelial cells, adipocytes, and fibroblasts, characterized by caveolins—integral membrane proteins that stabilize caveolae by inserting into the inner leaflet [167,168]. Caveolin-1 (Cav-1) is crucial for caveolae formation, associating with cholesterol and sphingolipids to form lipid domains enriched in signaling molecules. Caveolae-mediated endocytosis (CvME) is slower than CME, taking over 20 min [169], initiated by ligand binding to caveolar receptors. Cavins, such as PTRF/cavin-1, are essential for caveolae integrity, interacting with caveolins and lipids to maintain structure and facilitate vesicle formation.

#### 3.2.4. Clathrin- and Caveolae-Independent Endocytosis

Some CPPs utilize clathrin- and caveolae-independent pathways, which are less understood but critical for cellular uptake. These coat-free mechanisms can be dynamin-dependent—as in the IL-2 receptor using lipid rafts—or dynamin-independent, relying on actin polymerization or ARF proteins [162]. Flotillins induce membrane invaginations, facilitating endocytosis of cargo like GPI-anchored proteins [169]. These diverse pathways highlight the complexity of CPP internalization, influenced by peptide properties and cellular context.

Figure 3 illustrates the cellular uptake mechanisms of various CPPs currently employed in the treatment of different cancer types.

## 4. Discussion

The exploration of CPPs as a vehicle for targeted cancer therapies offers a promising path forward in addressing the significant challenges faced in oncology, particularly in overcoming drug resistance, improving the specificity of therapeutic delivery, and minimizing systemic toxicity. Throughout the body of research reviewed here, CPPs have demonstrated an impressive versatility, functioning both as delivery vehicles for chemotherapeutic agents, biologics, and nucleic acids and as independent therapeutic agents with intrinsic anticancer properties.

### 4.1. Dual Role of CPPs as Therapeutic Agents and Precision Delivery Systems in Oncology

Cell-penetrating peptides (CPPs) present a multifaceted strategy in oncology, merging delivery efficacy with direct therapeutic action. By facilitating precise transport of agents into cancer cells, CPPs enhance chemotherapeutic effectiveness, promote selective tumor imaging, and contribute directly as anticancer agents through mechanisms like apoptosis induction and p53 stabilization. This dual function enables CPPs to address complex oncogenic pathways and cellular targets, paving the way for innovative, multi-pronged cancer treatments that maximize tumor targeting while sparing healthy tissue.

One of the most compelling applications of CPPs is their ability to facilitate the delivery of diverse therapeutic agents directly into cancer cells. CPPs like PEP-010, ATX-101, and pVEC have proven particularly effective in this regard. For instance, PEP-010’s bifunctional sequence, which facilitates apoptosis by disrupting the caspase-9/PP2A interaction in breast cancer cells, exemplifies the potential of CPPs in targeting intracellular pathways that are otherwise difficult to access. Its ongoing clinical evaluation in combination with chemotherapeutics further underscores its therapeutic promise. Similarly, ATX-101 demonstrates the capacity of CPPs to enhance the efficacy of DNA-damaging agents by disrupting PCNA, a critical protein involved in DNA repair and cell survival, thus sensitizing cancer cells to chemotherapy.

Another notable example is AVB-620, a protease-cleavable peptide designed for tumor imaging. This peptide’s unique FRET-based mechanism offers real-time intraoperative tumor visualization, providing a significant advantage in surgical oncology by allowing for precise tumor resection. The success of AVB-620 highlights the broader potential of CPPs in diagnostic applications, expanding their utility beyond purely therapeutic roles.

These examples emphasize the dual capability of CPPs to function as both delivery vehicles and therapeutic agents, offering a versatile approach to overcoming one of the major hurdles in cancer treatment: ensuring the efficient and selective delivery of therapeutics to tumor cells while sparing healthy tissue.

Beyond their role as delivery vectors, several CPPs exhibit intrinsic therapeutic properties, acting directly as anticancer agents. p28, derived from Pseudomonas aeruginosa, is a standout example of a CPP that both penetrates tumor cells and modulates critical intracellular pathways. By stabilizing the tumor suppressor protein p53 and inducing cell cycle arrest, p28 has shown efficacy across several cancer types, including glioblastoma and hepatocellular carcinoma in both preclinical and clinical studies. Its ability to target both wild-type and mutant forms of p53 provides a broad therapeutic range, addressing one of the key challenges in cancer treatment: the frequent mutation or dysregulation of p53 in tumors.

Another remarkable peptide is melittin, derived from honeybee venom, which induces apoptosis in cancer cells by disrupting their membranes. Modified versions of melittin have improved its selectivity for tumor cells, reducing the risk of off-target toxicity while maintaining its potent anticancer effects. This highlights the potential of venom-derived CPPs in oncology, particularly in targeting TAMs and enhancing the efficacy of gene therapy through efficient intracellular delivery of siRNAs.

### 4.2. Structural Diversity and Mechanisms of Cellular Uptake

The structural diversity of CPPs, including cationic, amphipathic, and anionic peptides, enables them to exploit various cellular uptake pathways [7]. As discussed, CPPs enter cells through mechanisms such as macropinocytosis, clathrin-mediated endocytosis (CME), caveolae-mediated endocytosis (CvME), and, in some cases, direct membrane translocation. Each pathway offers distinct advantages depending on the cargo being delivered and the properties of the CPP itself. For example, cationic CPPs like pVEC efficiently bypass endocytic routes by directly translocating across the membrane, a feature that is particularly advantageous for delivering nucleic acid-based therapeutics as it mitigates the risk of endosomal entrapment.

The ability of CPPs to adopt different secondary structures in response to their environment further modulates their cellular uptake efficiency. Peptides like Pep-1 and MAP showcase how secondary structure can be leveraged to facilitate membrane destabilization and penetration. MAP’s amphipathic design, for instance, enables it to disrupt membrane integrity, making it effective not only as a delivery vehicle but also as a potent cytotoxic agent in its own right.

### 4.3. ADMET Challenges

While CPPs show remarkable potential as vectors for the targeted delivery of therapeutic cargos and as therapeutic agents in their own right, several critical challenges continue to constrain their clinical utility. The intrinsic peptide-based nature of CPPs renders them chemically unstable, making them vulnerable to proteolytic degradation by extracellular and intracellular enzymes such as proteases abundant in blood plasma, liver, and various tissues. This susceptibility to enzymatic cleavage can significantly reduce the concentration of active CPPs upon exposure to physiological environments, thereby diminishing their effectiveness at the intended site of action. To counteract rapid degradation, stabilized CPP variants and chemical modifications have been developed to extend CPP half-life and enhance their pharmacokinetic profile in vivo. Common strategies include the incorporation of non-natural amino acids, peptide cyclization, or PEGylation, each designed to reduce protease recognition and degradation rates. However, these modifications must be carefully optimized, as altering CPPs’ physicochemical properties can impact their membrane permeability and intracellular delivery efficiency.

A further challenge lies in achieving tissue-specific targeting and cellular selectivity. Although CPPs are adept at crossing cellular membranes, their limited specificity poses risks for off-target effects in non-target tissues, potentially resulting in undesirable side effects. To address this, current research efforts are focused on engineering CPPs with tailored sequences or incorporating specific ligands to improve binding affinity for target cells, such as cancer cells, thus enhancing specificity and reducing off-target interactions.

Another significant consideration is the immunogenicity and toxicity of CPPs. While strategies like PEGylation can reduce immunogenic responses, maintaining a balance between CPP stability and minimal immunogenicity remains challenging. This is particularly relevant in clinical applications where uncontrolled immune responses could compromise safety and efficacy. Ongoing research is investigating engineered CPP variants that minimize immune activation while preserving their functional properties.

Despite their general ability to permeate cell membranes, CPPs face limitations when penetrating deeper into solid tissues, which is a crucial factor in treating conditions within solid tumors or organs. Many CPPs lack the capability for efficient diffusion into dense tissues, hindering their effectiveness in such settings. Advances in CPP design are exploring modifications responsive to the unique microenvironmental conditions of tumors or leveraging secondary delivery systems to enhance tissue penetration.

Taken together, these challenges underscore the need for continued innovation in CPP engineering, balancing stability, selectivity, immunocompatibility, tissue penetration, and economic viability. Addressing these multifaceted issues will be essential for advancing CPPs toward more effective and clinically feasible therapeutic applications.

### 4.4. Future Directions

While CPPs hold great promise, several challenges remain in translating preclinical success to clinical practice. Key among these is the issue of selective targeting, as CPPs can sometimes lack specificity, leading to off-target effects. However, ongoing research into peptide modifications, such as the development of homing peptides like PEGA conjugated to pVEC, and the use of protease-cleavable linkers, as seen in AVB-620, is addressing this limitation by enhancing tumor specificity.

Moreover, the pharmacokinetic properties of CPPs, particularly their rapid renal clearance and potential for immunogenicity, present obstacles that must be overcome. Strategies such as the incorporation of unnatural amino acids, PEGylation, and the use of carrier proteins are being explored to improve the stability and bioavailability of CPPs, extending their half-life in circulation and enhancing their therapeutic efficacy.

## 5. Conclusions

CPPs offer a highly promising and innovative platform for cancer therapy, addressing critical limitations in targeted drug delivery. Their capacity to transport a wide range of therapeutic agents—including small molecules, proteins, and nucleic acids—directly into cancer cells without compromising the cargo’s functionality has positioned them as a key tool in next-generation oncological treatments. CPPs’ ability to bypass biological barriers, penetrate tumor tissues, and even deliver drugs into intracellular compartments highlights their potential to revolutionize cancer therapy, particularly for aggressive and drug-resistant cancers.

However, challenges remain in the clinical translation of CPP-based therapies. Key obstacles include variability in cellular uptake across different tumor types and the issue of endosomal entrapment, which can limit therapeutic efficacy by preventing payloads from reaching their intended intracellular targets. Despite these limitations, recent advances in CPP design, such as the incorporation of endosomolytic elements and improved peptide formulations like spaced oligoarginine conjugates, are showing promising results in overcoming these barriers. These innovations enhance the efficiency of endosomal escape and improve the precision of drug delivery to critical intracellular compartments, such as the nucleus.

Moreover, the refinement of CPP structures through the use of non-natural amino acids, hydrocarbon stapling, and multifunctional conjugates has led to significant improvements in peptide stability, bioavailability, and specificity. This opens new avenues for the development of combination therapies, where CPPs can be integrated with existing chemotherapeutic or immunotherapeutic agents to enhance efficacy and reduce off-target effects.

In conclusion, the evolving landscape of CPP research is successfully fostering their broader application in cancer treatment. By refining their structural and functional properties, CPPs hold immense potential for delivering precise, effective, and minimally toxic therapies. As clinical trials progress and new CPP formulations emerge, they are poised to play a pivotal role in overcoming the major challenges of cancer therapy, bringing us closer to more personalized and targeted treatment strategies for a range of malignancies.

## Figures and Tables

**Table 1:** Summary of the properties, mechanism, functions, and clinical trials of the CPPs with cancer-targeting applications discussed in Section 2.

CPP	Type	Cancer Types	Function	Mechanism	Clinical Trial ID	References
PEP-010	Cationic	Breast cancer	Restores apoptotic pathways	Disrupts the caspase-9/PP2A interaction, activating caspase-dependent apoptosis	NCT04733027 (Phase I)	[23,26,28]
ATX-101	Cationic	Multyple myeloma/Sarcoma	Reaches the cell nucleus to enhance damage repair, cellular stress response, and the efficacy of several anticancer agents	Disrupts the PCNA/APIM-containing protein interaction	NCT05116683 (Phase II), NCT04814875 (Phase I/II), NCT01462786 (Phase I)	[30,31,32]
AVB-620	Cationic	Breast cancer	Real-time tumor visualization during surgery	CPP conjugated with fluorophores Cy5/Cy7 for FRET, targeted to human breast cancer cells due to their MMP overexpression	NCT02391194 (Phase I), NCT03113825 (Phase II)	[37]
Z12 and ZEBRA-Derived CPPs	Cationic	Broad spectrum, including aggressive brain cancers	Component of cancer vaccines	Promotes immune responses against tumors when conjugated with multi-epitopic antigens	NCT04046445 (Phase I)	[38,39]
pVEC and PEGA	Cationic	Breast cancer	Targeted drug delivery vector	Selective non-endocytic translocating mechanism (pVEC) by targeting molecular markers on tumor cells when conjugated with homing peptides (PEGA)		[38,46,47]
Pep-1	Cationic	Broad spectrum	Targeted macromolecular carrier and drug delivery vector	High selective non-endocytic translocation through cancer cell membranes is primarily due to the high presence of acidic components		[55,56]
MAP	Cationic	Broad spectrum	Bifunctional CPP that disrupts cancer cell membranes	Selective strong electrostatic interactions with negatively charged phospholipids		[73]
p28	Cationic	Multiple cancer types, including glioblastoma and hepatocellular carcinoma	Promotes cell-cycle arrest and apoptosis in tumor cells	Interacts with wild-type and mutant p53 proteins, inhibiting their ubiquitination and regulating their levels	NCT00914914 (Phase I), NCT01975116 (Phase I), NCT05359861 (Phase II), NCT06102525 (Phase I)	[77,78,79,80]
SAP and SAP(E)	Proline-rich amphipatic	Broad spectrum	Targeted drug delivery vector with minimal toxicity	Specific electrostatic interactions with negatively charged membrane components (SAP); internalization of aggregates in a non-clathrin or caveoline-mediated endocytosis (SAP(E))		[84,85]
Bac1-24	Proline-rich amphipatic	Broad spectrum, particularly solid tumors	Targeted delivery agent of therapeutic proteins and peptides	Hydrophobic domains and specific electrostatic interactions with negatively charged phospholipids		[89]
BIM-SAHB_*A*_	Stapled peptide	Hematologic cancers	Restores apoptosis in resistant cancer cells	Blocks the anti-apoptotic sequestration of BAX/BAK BH3 helices, mimicking the BH3 death domain		[90]
SAHB_*D*_	Stapled peptide	Cancers where MCL-1 overexpression is a critical survival factor (myeloma, acute myeloid leukemia, melanoma, etc.)	Restores apoptosis in resistant cancer cells	Inhibits the MCL-1 anti-apoptotic activity, disrupting its interaction with pro-apoptotic proteins		[91]
ALRN-6924	Stapled peptide	Broad spectrum, including breast cancer and acute myeloid leukemia	Restores p53 function, reactivating apoptosis	Binds strongly to MDM2 and MDMX, inhibiting the p53 suppression	NCT02264613 (Phase I/II), NCT04022876 (Phase I), NCT03654716 (Phase I), NCT05622058 (Phase)	[97,98]
P1pal-7	Pepducin	Breast, lung, and ovarian cancer	Reduces tumor growth and slows cancer progression. Anti-angiogenic agent	Interacts with PAR1, inhibiting its activation		[103,104]
EN1-iPeps	Homeodomain-derived	Breast cancer	Triggers a selective apoptosis response	Inhibits the EN1 transcription factor in tumor cells where it is overexpressed		[111,113]
Vectocell®/DPVs	HS Binding CPP	Broad spectrum	Targeted drug delivery agent (from small compounds to macromolecules)	Caveolae-mediated endocytosis of DPVs-Glycosaminoglycan clusters		[115]
CPPecp	HS Binding CPP	Tumors with high HS expression, including colon cancer	Inhibits cancer cell migration and angiogenesis	Binding to overexpressed heparan sulfate on the surface of cancer cells		[116]
Melittin and derivatives	Derived from animal venoms and toxins	Broad spectrum	Drug delivery vector and apoptosis inductors in tumor-associated macrophages	An amphipathic α-helix structure enables interactions with the membrane, allowing the internalization of conjugated pro-apoptotic peptides		[92,119,120]
Lycosin-I and R-lycosin-I	Derived from animal venoms and toxins	Broad spectrum	Induces apoptosis in cancer cells and inhibits cell proliferation	Activates the mitochondrial death pathway and upregulates p27		[125,126,127,128]
Pardaxins	Derived from animal venoms and toxins	Broad spectrum including aggressive cancers such as ovarian cancer and oral squamous cell carcinoma	Apoptosis inductor	Generation of ROS and mitochondrial membrane depolarization		[134,139]
BT1718	Cyclic	Solid and refractary tumors	Selective realese of cytotoxic agents	Binds to overexpressed MT1-MMP in tumors realising DM1, a cytotoxic payload	NCT03486730 (Phase I/II)	[142,144,145]
^177^Lu-DOTA^0^-Tyr^3^-Octreotate (Lutathera®)	Cyclic	SSTR2-positive neuroendocrine tumors	Selective delivery of cytotoxic agent	This radioconjugate utilizes the somatostatin analog TATE to target SSTR2-positive neuroendocrine tumors, delivering a cytotoxic dose of β radiation	NCT02125474 (Phase II), NCT02236910 (Phase II), NCT03325816 (Phase I/Phase II)	[146,150,151]

## Data Availability

Not applicable.

## Abbreviations

The following abbreviations are used in this manuscript:

ADMET	Absortion, Distribution, Metabolism, Excretion and Toxicity
AML	Acute Myeloid Leukemia
AMP	Antimicrobial Peptide
BTC	Bicycle Toxin Conjugate
CME	Clathrin-Mediated Endocytosis
CPP	Cell-Penetrating Peptide
cCPP	Cationic Cell-Penetrating Peptide
CvME	Caveolae-Mediated Endocytosis
DPV	Diatos Peptide Vector
ELP	Elastin-Like Polypeptide
EN1	Engrailed 1
ER+	Estrogen Receptor-Positive
FRET	Fluorescence Resonance Energy Transfer
GAG	Glycosaminoglican
GEP-NET	Gastroenteropancreatic Neuroendocrine Tumor
GPCR	G Protein-Coupled Receptor
HS	Heparan Sulfate
HTH	Helix-turn-helix
iPep	Interfering Peptide
MAP	Model Amphipathic Peptide
MDR	Multidrug Resistance
MMP	Matrix Metalloproteinase
MT1-MMP	Membrane Type 1 Matrix Metalloproteinase
NET	Neuroendocrine Tumor
OSCC	Oral Squamous Cell Carcinoma
PAR1	Protease-Activated Receptor 1
PCNA	Proliferating Cell Nuclear Antigen
PDX	Patient-Derived Xenograft
PGE2	Prostaglandin E2
PNA	Peptide Nucleic Acid
PP2A	Protein Phosphatase 2A
PRRT	Peptide Receptor Radionuclide Therapy
PS	Phosphatidylserine
ROS	Reactive Oxygen Species
saCPP	Secondary Amphipathic Cell-Penetrating Peptide
SAR	Structure-Activity Relationship
SSTR2	Type 2 Somatostatin Receptor
TAM	Tumor-Associated Macrophage
TNBC	Triple-Negative Breast Cancer
WT	Wild-Type
